# Parkinson’s Disease and Metal Storage Disorders: A Systematic Review

**DOI:** 10.3390/brainsci8110194

**Published:** 2018-10-31

**Authors:** Edward Botsford, Jayan George, Ellen E. Buckley

**Affiliations:** 1 University of Sheffield Medical School, Beech Hill Road, Sheffield S10 2RX, UK; 2 General Surgical Department, Sheffield Teaching Hospitals NHS Foundation Trust, Herries Road, Sheffield S5 7AU, UK; jayan.george@aol.com; 3 University of Sheffield, Western Bank, S10 2TN Sheffield, UK; 4 Sheffield Institute for Translational Neuroscience, University of Sheffield, 385a Glossop Road, Sheffield S10 2HQ, UK; e.e.buckley@sheffield.ac.uk; 5 INSIGNEO Institute for in silico Medicine, University of Sheffield, Pam Liversidge Building, Sheffield S1 3JD, UK

**Keywords:** Parkinson’s disease, Parkinsonism, metal storage disorders, inborn error of metabolism

## Abstract

Metal storage disorders (MSDs) are a set of rare inherited conditions with variable clinical pictures including neurological dysfunction. The objective of this study was, through a systematic review, to identify the prevalence of Parkinsonism in patients with MSDs in order to uncover novel pathways implemented in Parkinson’s disease. Human studies describing patients of any age with an MSD diagnosis were analysed. Foreign language publications as well as animal and cellular studies were excluded. Searches were conducted through PubMed and Ovid between April and September 2018. A total of 53 publications were identified including 43 case reports, nine cross-sectional studies, and one cohort study. The publication year ranged from 1981 to 2018. The most frequently identified MSDs were Pantothenate kinase-associated neurodegeneration (PKAN) with 11 papers describing Parkinsonism, Hereditary hemochromatosis (HH) (7 papers), and Wilson’s disease (6 papers). The mean ages of onset of Parkinsonism for these MSDs were 33, 53, and 48 years old, respectively. The Parkinsonian features described in the PKAN and HH patients were invariably atypical while the majority (4/6) of the Wilson’s disease papers had a typical picture. This paper has highlighted a relationship between MSDs and Parkinsonism. However, due to the low-level evidence identified, further research is required to better define what the relationship is.

## 1. Introduction

Parkinson’s disease (PD) is a common and debilitating neurodegenerative disorder. First described in 1817 by James Parkinson, PD is a chronic condition distinguished by bradykinesia, rigidity, postural instability, and resting tremor often described as “pill-rolling.” The clinical features are due to the loss of dopaminergic neurones located in the pars compacta of the substantia nigra. Why these neurons are lost is poorly understood. However, numerous studies from animal models and familial cases of PD have identified that accumulation of cytoplasmic inclusions of alpha-synuclein (α-synuclein) called Lewy bodies, oxidative stress, and mitochondrial dysfunction may all play a pathogenic role in their destruction [1,2]. 

Despite multiple well-documented risk factors suggesting an environmental association such as well-water drinking, pesticide exposure, head injury, and rural living [3], only increased age carries sufficient statistical evidence to be causative [4]. Male gender and Caucasian ethnicity were reported to increase the risk of PD in research studies while tobacco smoking has been found to be protective. Therefore, the existence of other aetiological mechanisms not yet identified must be considered.

Parkinsonism is a clinical picture of a tremor, rigidity, bradykinesia, and postural instability most frequently caused by sporadic PD. However, it has been described in many other conditions. Parkinsonism can be associated with additional features such as dystonia, early autonomic dysfunction, a rapidly progressive disease course, or levodopa unresponsiveness [5]. In this instance, it is described as atypical Parkinsonism since it differs from the typical clinical picture seen in Parkinson’s disease.

Inborn errors of metabolism (IEMs) are a large collection of individually rare but collectively common inherited conditions [6]. They are a diverse set of conditions that occur as a result of a monogenic mutation resulting in a deficiency of an enzyme or cofactor [7]. Metal storage disorders are a large subset of these. 

Studies have found that patients with PD have increased levels of iron accumulation in the basal ganglia compared with healthy controls [8]. Research has also been conducted into the potential toxic mechanisms of iron causing nigral cell death and leading to PD features in sporadic PD patients even though it remains unclear whether neuronal death is a direct result of iron accumulation or if the accumulation is a by-product of dopaminergic cell death [9]. 

This systematic review aims to identify whether there is a wider pathological link between PD and metal storage disorders by exploring published accounts of Parkinsonism in patients with a previously diagnosed metal storage disorder. Identifying other conditions that produce Parkinsonian-like clinical features may uncover novel pathological mechanisms that contribute to the development of PD. In addition, this paper will discuss whether the clinical features seen in the patients with metal storage disorders displaying Parkinsonism are of a typical picture seen in PD or if they are more similar to atypical Parkinsonism.

## 2. Materials and Methods

This systematic review was conducted by following the Preferred Reporting Items for Systematic Reviews and Meta-Analyses (PRISMA) 2009 guidance [10].

### 2.1. Search Terms

Systematic literature searches were conducted in the PubMed and Ovid SP databases including all published articles prior to the search date. The last search was completed on 6 September, 2018. The titles and abstracts were searched by combining two search terms (Term A and Term B). Term A was ‘Parkinson,’ ‘Parkinson’s,’ ‘Parkinsonism,’ or ‘Parkinsonian’ while Term B included each of the individual metal storage disorders. A list of metal storage disorders was collated from a relevant review article [11]. A full list of the search terms can be found in Appendix A. 

### 2.2. Inclusion Criteria

Human studies of all designs were considered except review articles. Only publications describing patients with a definite genomic or biochemical diagnosis of a metal storage disorder were analysed. Publications reporting adults, children, and infants were all included since metal storage disorders and Parkinsonian features can present at any age. Cohorts from all nationalities and ethnic backgrounds were also included. 

### 2.3. Exclusion Criteria

Animal and cellular model studies were excluded as well as autopsy reports. Papers describing PD patients with MSD associated gene mutations were also excluded unless they had a confirmed diagnosis of that disorder. Publications written in languages other than English, without whole text translations available, were excluded. Reviews and letters to editors were also excluded but the references examined to identify any potentially relevant references that the searches had omitted were accepted. 

### 2.4. Selection Process

The publications acquired from the searches were screened in line with the selection criteria by reading the titles and abstracts to assess relevance. Afterward, full texts were sought for all papers eligible for inclusion. Two reviewers conducted this screening process to ensure adherence to selection criteria. Conflicts were resolved through discussion between the reviewers.

### 2.5. Data Extraction

The primary outcomes extracted from the publications were the type of study, the IEM affecting the patients reported in the study, and whether the patient had features of typical or atypical Parkinsonism. Patients were identified as possessing atypical Parkinsonism if there was evidence of early autonomic dysfunction, a rapidly progressive course, lack of asymmetrical features at onset, and a poor response to conventional levodopa therapy or a Parkinson-Plus syndrome, as per the definition in the introduction. Where available, the gender, age of onset of Parkinsonian features, smoking status, and ethnicity were recorded as secondary outcomes. A breakdown of the data collected from each individual paper including the clinical picture of the patients can be found in Table A1.

## 3. Results

**Figure 1:**
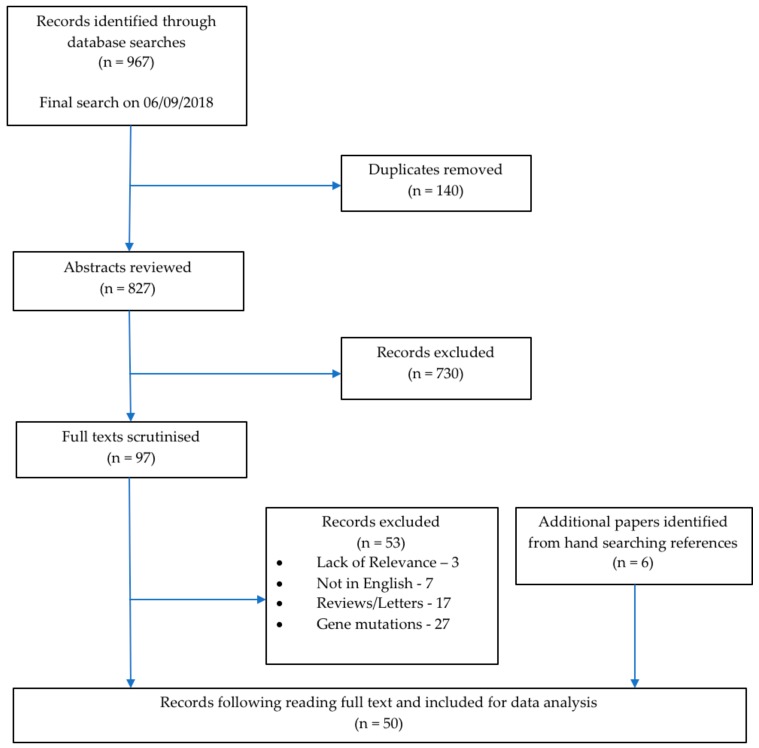
Prisma flow chart illustrating the search strategy and the selection process.

In total, 967 publications were identified corresponding to 827 unique articles which underwent the screening process. Following the title and abstract review, 730 were excluded since they did not satisfy the inclusion criteria. Following full text screening, a further 53 records were excluded. Six additional relevant publications were identified from hand searching the reference lists of the reviews and letters identified in the searches. A total of 50 papers were included in this review. Figure 1 shows a PRISMA flowchart of the selection process. The final group of articles consisted of 40 (80.0%) case reports and series, nine (18.0%) cross-sectional studies, and one (2.0%) cohort study (Table 1). The year of publications ranged from 1981 to 2018 with three (6.0%) papers published before 1990, three (6.0%) papers published in the decade between 1991 to 2000, 12 (24.0%) papers published from 2001 to 2010, and 32 (64.0%) papers published in the current decade from 2011 to 2018 (Table 1).

Typical Parkinsonism was reported in 16 (32.0%) publications and atypical in 38 (76.0%) publications, which is shown in Table 2. Of these papers, four described subjects with both typical and atypical features. Additionally, 173 patients were reported to have Parkinsonism, 86 (49.7%) were male, and the average age of onset was 35 years old. The ratio of males to females observed was 0.99:1 (86 males to 87 females). The smoking status was not reported in any of the publications (Appendix B).

Pantothenate kinase-associated neurodegeneration (PKAN), which is the most prevalent neurodegenerative brain iron accumulation (NBIA) disorder, was the most documented metal storage disorder and was reported in 11 papers. All of these publications described patients displaying features of atypical Parkinsonism. Three papers also described subjects with typical Parkinsonian features. Within the 85 PKAN patients reflected by these articles, the mean age of onset of Parkinsonism was 33 years old. The gender ratio was 1.36:1 with 49 males and 36 females described. PLA2G6-associated neurodegeneration (PLAN) was another frequently identified NBIA with three publications identified. Typical parkinsonism features were described by two of these papers while the remaining publications reported atypical Parkinsonism. Other NBIAs identified beta-propeller protein-associated neurodegeneration (BPAN) with five publications (four with atypical Parkinsonism and one with typical features), Kufor-Rakeb Syndrome with five articles (all atypical, although one described patients with typical features), and mitochondrial-membrane protein-associated neurodegeneration (MPAN) (three papers describing atypical parkinsonism). In addition, three publications described atypical features in subjects with neuroferritinopathy and one paper described a patient with Aceruloplasminemia presenting with features of atypical Parkinsonism. An additional paper described a subject with atypical features who suffered from an unknown type of NBIA. 

After PKAN and NBIAs, the next most reported metal storage disorders were Hereditary Haemochromatosis (HH) and Wilson’s disease. Seven articles were identified that reported patients with HH and four papers (57.1%) described typical Parkinsonism. In these publications, 14 subjects were described including 10 males and 4 females (a ratio of 2.5:1). The mean age of onset of Parkinsonism in these patients was calculated at 53 years of age. Parkinsonism in Wilson’s disease patients was reported in six papers in which four (66.6%) described typical features while the remaining two papers (33.3%) displayed atypical pictures. The mean age of onset in the patients described was 46 years of age and a gender ratio 0.75:1 (three males and six females). 

## 4. Discussion

Parkinsonian presentation in patients with metal storage disorders is an area of growing interest. The number of publications identified in this study increases each decade. While only three articles were published earlier than 1990, between the years 2010 to 2018, 29 papers were identified. This demonstrates an increasing amount of research being conducted in this field and a growing appreciation for a possible correlation between Parkinsonism and metal storage disorders. 

The family of neurodegenerative brain iron accumulation (NBIA) disorders includes Pantothenate kinase-associated neurodegeneration (PKAN), Aceruloplasminemia, beta-propeller protein-associated neurodegeneration (BPAN), Kufor-Rakeb Syndrome, mitochondrial-membrane protein-associated neurodegeneration (MPAN), neuroferritinopathy, and PLA2G6-associated neurodegeneration (PLAN). Articles discussing all of these disorders were identified by our searches and described patients displaying Parkinsonism. These all showed a similar phenotype with young average ages of onset of Parkinsonism ranging from 13 years old (Kufor-Rakeb syndrome) to 61 years old (neuroferritinopathy) and predominantly atypical Parkinsonian features. This reflects the similar pathologies across the NBIA family of disorders. In all NBIAs, increased deposition of iron in brain tissue is observed. It is unclear whether this deposition is the direct cause of neurodegeneration or if it is simply a marker of the degeneration occurring as a result of some other pathological mechanism. However, Parkinsonism as well as dystonia appears well documented across all NBIAs. 

In line with our findings, PKAN is the most common NBIA accounting for roughly half of all cases [12]. In the 11 publications describing PKAN, all papers described patients with atypical features while three also described patients with typical Parkinsonism. The atypical features displayed in these patients were a poor levodopa response [13,14,15,16], a lack of asymmetrical features [15,17,18,19], or the presence of dystonia in addition to Parkinsonism [14,20,21,22] (Appendix B). In two publications, pyramidal signs were also observed [19,23]. Recent research has established that Lewy body pathology is not observed in PKAN, which may explain why atypical features of Parkinsonism are more commonly seen [24]. Historical reports of patients with PKAN have found α-synuclein inclusions in neurons [7,25]. However, Schneider et al. believe these patients may have been misdiagnosed since these reports were published before gene identification was available for diagnosing PKAN [24]. They describe a recent series of genetically confirmed PKAN patients in which all lacked any evidence of Lewy body pathology. This suggests a differing pathology is occurring in these patients. Our results showing a high prevalence of atypical Parkinsonism in PKAN sufferers supports this hypothesis. At the same time, the widespread presence of α-synuclein inclusions in the central nervous system (CNS) tissue of PLAN patients is well documented [26,27], which indicates a potential pathological link between PLAN and sporadic PD. The results from this review support this link with two of the three publications describing patients with PLAN due to features of typical Parkinsonism [28,29]. In the one paper describing atypical parkinsonism features in patients with PLAN, dystonia was present [30]. 

Hereditary haemochromatosis (HH) was also frequently identified in this systematic review. Four papers reported typical Parkinsonism [31,32,33,34] while atypical features were described in the remaining three papers. All papers were related to unresponsiveness to levodopa [35,36,37]. These reports of Parkinsonism and HH presenting concurrently indicate that research into iron accumulation in the CNS tissue of HH patients may clarify the pathological link between HH and PD. The pathological processes and brain regions involved in HH are not well understood. In particular, the location of iron accumulation in CNS tissue is poorly documented. Since Parkinson’s disease treatments were reported to be ineffective in these patients and an atypical picture was observed, it may be that a different area of the brain is affected. Further research is required in order to identify how and where the iron accumulation occurs in order to draw further conclusions from this result.

Six publications described Parkinsonism in Wilson’s disease. It is well established that copper deposition, as seen in Wilson’s disease patients, commonly has toxic effects in the brain, which leads to severe neurological features [38,39]. How copper causes neuronal death is not well understood even though it is generally accepted that the copper accumulates extracellularly and does not enter neurons [38]. Within this group, four publications described typical Parkinsonism in Wilson’s disease [40,41,42,43] and two described atypical parkinsonism [44,45]. Although Parkinsonism is a common feature of neurologic Wilson’s disease [38,39], there is no evidence to suggest that Wilson’s disease causes Lewy body pathology. Despite this, all of the Wilson’s Disease patients from this study displayed levodopa responsiveness. This included two patients with atypical parkinsonism where one had dystonia [44] and one had epilepsy [45]. As mitochondrial dysfunction plays a large role in the pathophysiology of PD [1,2], the extracellular accumulation of copper in the CNS may have the same effect on mitochondria within the neuron that it does within the hepatocytes. Despite the similarities in clinical features and the response to levodopa, these patients’ demographics differ significantly to those seen in the sporadic PD population, which is outlined by Rizek et al. [46]. The average age of onset of Parkinsonism in these Wilson’s disease patients was reported as 46 years old, which is considerably younger than in the sporadic PD population (mean age 65 years old). Furthermore, twice as many females as males were described as having Parkinsonism, which differs greatly to the ratio of 1.5 males per females seen in the sporadic PD population. However, since only nine patients were described, this is not a large enough population to draw generalizable conclusions especially since the lack of α-synuclein pathology indicates the presence of a different pathological process. 

Despite previous research establishing that cigarette smoking is protective for PD [4], the smoking status was not reported in any of the publications (Appendix B). Therefore, it was not possible to investigate this in the current study. It would be pertinent to include the smoking status in the patient demographics of all future publications describing PD or features of Parkinsonism.

The precise nature of the relationship between iron accumulation in patients with Parkinsonism is not clear. Autopsy studies were excluded from this review since they offered retrospective details of the clinical picture and the timelines were poorly outlined. However, they could yield some useful findings in patients with metal storage disorders. Post mortem brain studies on patients with NBIAs allows us to investigate the correlation between the quantity of iron and the severity of PD features. Should this confirm that iron accumulation in the brain leads to the development of Parkinsonism, it follows that treatments to reduce the CNS iron levels, or act as an iron chelator, could be developed as an early treatment for patients with sporadic PD in order to delay the Parkinsonian features.

An important factor to consider when interpreting these results is the level and quality of evidence available in the literature. The majority of the publications included were case reports and case series that the Oxford Centre of Evidence-Based Medicine considers level 4 evidence [47]. However, due to the rarity of these individual IEMs, this was the highest level of evidence available. Case reports can be subject to bias and, although no formal bias assessment was conducted, a more favourable response to levodopa or exaggeration of the severity of the features may have been reported. This was taken into consideration when evaluating articles for inclusion and any paper describing a response less than moderate to levodopa was classed as atypical. 

## 5. Conclusions

In conclusion, the presence of Parkinsonism in metal storage disorders is an under reported topic. Establishing the relationships between these conditions may clarify the pathological mechanisms of Parkinsonism. Therefore, it is a field of growing interest with the number of publications describing patients with metal storage disorders displaying Parkinsonism growing substantially each decade. This review has demonstrated the following:

1. There is evidence of Parkinsonism coexisting with metal storage disorders in particular neurodegenerative brain iron accumulation disorders.
2. Patients with these metal storage disorders have an earlier age of onset of Parkinsonism than sporadic PD patients, which suggests additional underlying pathological processes are taking place. The ratio of males to females seen in many of these also differs significantly to the sporadic PD population, which further indicates a differing pathogenesis.
3. Future research must be conducted at a higher level than individual case reports to better assess the relationship between metal storage disorders and Parkinsonism. Cohort studies or case control studies using large cohorts will lead to a reliable dataset. At the same time, research in sporadic PD patients will identify whether any of the pathological mutations or processes are involved in the disorders discussed in relation to the development of Parkinsonism.
4. Smoking status and ethnicity should be documented in all future studies of Parkinsonism since Caucasian ethnicity is a large risk factor in sporadic PD while cigarette smoking appears to be protective. Recording these demographics will allow for the investigation of their presence in patients with metal storage disorders.

## Figures and Tables

**Table 1:** Characteristics of included publications.

Year of Publication Range	1981–2018
**Number of Publications per Decade**	
Before 1990	3
1991–2000	3
2001–2010	12
2011–2018	32
**Type of Study**	
Cohort study	1
Cross-sectional study	9
Case reports/series	40

**Table 2:** Characteristics of the disorder-related parkinsonism described in the included publications, in order of the number of papers identified.

Condition	Metal Involved	Brain Region Implicated	Total No. of Papers (No. Typical, No. Atypical)	No. (% Total) of Male and Female Patients Described	Average Age of Patients (Years, Mean ± Standard Error)
Panthonase Kinase associated Neurodegeneration (PKAN)	Iron	Basal ganglia (GP, SN)	11 (3;11)	49 M (57.6%) 36 F (42.4%)	33 ± 3.8
Hereditary Haemochromatosis	Iron	-	7 (4;3)	10 M (71.4%) 4 F (28.6%)	53 ± 3.3
Wilson’s Disease	Copper	Basal ganglia (PMN, GP)	6 (6;4)	3 M (33.3%) 6 F (66.6%)	46 ± 6.8
Beta-Propeller Protein-Associated Neurodegeneration (BPAN)	Iron	Basal ganglia (SN, GP)	5 (1,4)	3 M (9.7%) 28 F (90.3%)	27 ± 1.1
Kufor-Rakeb Syndrome	Iron	Basal Ganglia (SN, GP)	5 (1;5)	10 M (90.9%) 1 F (9.1%)	13 ± 0.7
Mitochondrial-Membrane Protein-Associated Neurodegeneration (MPAN)	Iron	Basal Ganglia (SN, GP)	3 (0;3)	1 M (50.0%) 1 F (50.0%)	25 ± 10.0
Neuroferritinopathy	Iron	Cerebellum, Basal ganglia, motor cortex	3 (0;3)	5 F (100.0%)	61± 17.5
PLA2G6-Associated Neurodegeneration (PLAN)	Iron	Basal ganglia (SN, GP)	3 (2;1)	3 M (60.0%) 2 F (40.0%)	24 ± 5.2
Pseudohypoparathyroidism	Calcium	Basal ganglia, deep white matter	3 (0;3)	1 M (33.3%) 2 F (66.6%)	43 ± 11.8
Fahr Disease	Calcium	Basal ganglia, deep white matter, cerebellum	2 (1;1)	1 M (50.0%) 1 F (50.0%)	56 ± 6.0
Aceruloplasminemia	Iron	Basal ganglia	1 (0;1)	4 M (80.0%) 1 F (20.0%)	NA
Neurodegenerative Brain Iron Accumulation (NBIA), Unknown Type	Iron	-	1 (0;1)	1 M (100.0%)	73 ± 0.0
**Total**	-	-	50 (16;38)	86 M (49.7%) 87 F (50.3%)	35 ± 1.6

GP = Globus Pallidus, F = Female, M = Male, PMN = Putamen, SN = Substantia Nigra, NA = Not Available.

## Supplementary Materials

Supplementary File 1

## Appendix A

Search terms. Term A: Parkinson, Parkinson’s, Parkinsonism, Parkinsonism. Term B: Aceruloplasminemia, Acrodermatitis, Bartter disease, BPAN, Calcium metabolism, CoPAN, Copper metabolism, DiGeorge, Fahr, Hemochromatosis, Hereditary rickets, Iron metabolism, Kufor-Rakeb syndrome, Magnesium metabolism, Menkes, Mitochondrial membrane protein-associated neurodegeneration, Neurodegenerative brain iron accumulation, Neuroferritinopathy, PLAN, PLA2G6-associated neurodegeneration, Phosphate metabolism, Pseudohypoparathyroidism, Tumoral calcinosis, Vitamin D metabolism, Wilson, Woodhouse-Sakati syndrome, Zinc metabolism.

## Appendix B

**Table A1:** Table showing the individual breakdown of the included publications. NA = Not Available.

Paper	Type of Paper	Condition	Male/Female	Average Age at Onset of Parkinsonism (years)	Ethnicity	Smoking Status	Typical Parkinsonism	Atypical Parkinsonism	Parkinsonism Features
Alberca, R. et al., 1987. [20]	Case report	PKAN	1M/1F	27	NA	NA	✓	✓	Female siblings: Typical features. Male sibling: associated with dystonia. Fast progression.
Batla, A. et al., 015. [48]	Case report	Neuroferritinopathy	1F	79	NA	NA		✓	Associated with dystonia.
Behrens, M.I. et al., 2010. [49]	Case Series	Kufor-Rakeb Syndrome	4M/1F	NA	Chilean	NA		✓	Parkinsonian features in all five pts. No tremor present. Supranuclear gaze palsy in 4/5, poor L-dopa response
Bozi, M. et al., 2009. [23]	Case report	PKAN	1M	15	NA	NA		✓	Mildly affected but associated with pyramidal signs.
Chinnery, P.F. et al., 2007. [50]	Cross-sectional study	Neuroferritinopathy	3F	NA	2 English, 1 French	NA		✓	Associated with dystonia in all three. No tremor present.
Costello, D.J. et al., 2004. [31]	Case report	Hereditary Haemochromatosis	3M/1F	53	NA	NA	✓		Four pts all with HH and IPD diagnoses, classical signs. Good L-dopa response.
Crosiers, D. et al., 2011. [51]	case report	Kufor-Rakeb syndrome	1M	10	Afghan	NA		✓	Associated with dystonia.
Czlonkowska, A. et al., 2018. [40]	Cross-sectional study	Wilson’s disease	NA	NA	Polish	NA	✓		Parkinsonism found in 11.3% (6/53 pts).
Darling, A. et al., 2017. [21]	Cross-sectional study	PKAN	22M/25F	NA	NA	NA		✓	Features of Parkinsonism displayed in all 47 pts. Associated with Dystonia.
Demarquay, G. et al., 2000. [35]	Case report	Hereditary Haemochromatosis	2M/1F	56	NA	NA		✓	Bradykinesia and rigidity on left side. Poor L-dopa response.
Di Fonzo, A. et al., 2007. [52]	Cross-sectional study	Kufor-Rakeb Syndrome	3M	NA	NA	NA	✓	✓	Features of Parkinsonism in all three pts. Supranuclear gaze palsy and hallucinations/psychotic episodes in 1/3, psychotic episodes in 1/3, and typical features in 1/3.
Diaz, N., 2013. [13]	Case report	PKAN	1F	NA	NA	NA		✓	L-dopa unresponsive, symmetrical features.
Eiberg, H. et al., 2012. [53]	Case report	Kufor-Rakeb Syndrome	1M	12	NA	NA		✓	Supranuclear gaze palsy, cognitive impairment, and hallucinations.
Evans, B.K. & Donley, D.K., 1988. [54]	Case report	Pseudohypoparathyroidism	1F	20	NA	NA		✓	Rest tremor and bradykinesia with mental retardation.
Fekete, R., 2012. [55]	Case report	NBIA, unknown type	1M	73	NA	NA		✓	Typical features. Poor L-dopa response but dystonia present upon removal of L-dopa.
Fonderico, M. et al., 2017. [56]	Case report	BPAN	1F	26	NA	NA	✓		Mild typical parkinsonism.
Gasca-Salas, C. et al., 2017. [44]	Case report	Wilson’s Disease	1F	38	NA	NA		✓	Tremor, clumsiness, rigidity, and dystonia in left arm. Good L-dopa response.
Giri, A. et al., 2016. [28]	Case report	PLAN	1F	27	NA	NA	✓		Typical Features, PD diagnosis.
Girotra, T., Mahajan, A. & Sidiropoulos, C., 2017. [32]	Case report	Hereditary Haemochromatosis	1M	41	Caucasian	NA	✓		Typical features, mild but clear response to L-dopa.
Gondim, F. de A.A. et al., 2014. [41]	Case Series	Wilson’s disease	2M/2F	28	Brazil	NA	✓		Four pts with typical features, all responded well to L-dopa.
Gore, E. et al., 2016. [57]	Case report	MPAN	1M	35	Kuwaiti	NA		✓	Early behavioural change.
Hayflick, S.J. et al., 2013. [58]	Cohort study	BPAN	3M/18F	25	NA	NA		✓	Developmental delay, dystonia, and parkinsonism. L-dopa responsive.
Hermann, A. et al., 2017. [59]	Case report	BPAN	1F	24	German	NA		✓	Supranuclear gaze palsy, dystonia, and no L-dopa response.
Ichinose, Y. et al., 2014. [60]	Case report	BPAN	1F	30	NA	NA		✓	Associated with dystonia.
Kim, Y.J. et al., 2015. [30]	Case Series	PLAN	1M/1F	14	Korean	NA		✓	Associated with dystonia in 2/2 pts.
Klysz, B., Skowronska, M. & Kmiec, T., 2014. [61]	Case report	MPAN	1F	15	NA	NA		✓	Chorea, dystonia, and psychological manifestations.
Kumar, N. et al., 2016. [33]	Case Series	Hereditary Haemochromatosis	2M/1F	59	1 Irish-Portuguese, 1 Scottish, 1 unknown	NA	✓		Parkinsonian signs in three pts. One responded well to L-dopa, one not treated.
Lee, C.-H. et al., 2013. [17]	Case report	PKAN	2M	20	Taiwanese	NA	✓	✓	Typical parkinsonism in one pt though onset at 18. Bilateral features in the other.
Lee, J.-H. et al., 2016. [14]	Cross-sectional study	PKAN	6M	36	NA	NA		✓	Poor response to L-dopa in all. Associated with dystonia in 4/6 pts, isolated parkinsonism in 2/6 pts.
Mak, C.M. et al., 2011. [18]	Case report	PKAN	1M	27	Hong Kong	NA		✓	Bilateral features.
Ni, W. et al., 2016. [62]	Case report	Neuroferritinopathy	1F	44	NA	NA		✓	No response to L-dopa, pyramidal signs.
Nielsen, J.E., Jensen, L.N. & Krabbe, K., 1995. [34]	Case report	Hereditary Haemochromatosis	1M	29	NA	NA	✓		Typical PD features, immediate improvement with L-dopa.
Nishioka, K. et al., 2015. [63]	Cross-sectional study	BPAN	7F	32	NA	NA		✓	Cognitive dysfunction as presenting symptom in all seven. Otherwise typical parkinsonism. L-dopa responsive.
Oder, W. et al., 1991. [42]	Cross-sectional study	Wilson’s Disease	NA	NA	NA	NA	✓		8/25 pts with parkinsonian features. Bradykinesia, resting tremor present.
Olgiati, S. et al., 2017. [64]	Cross-sectional study	MPAN	NA	NA	NA	NA		✓	9/15 pts with parkinsonian features. Cognitive impairment and pyramidal signs seen.
Pearson, D.W. et al., 1981. [65]	Case report	Pseudohypoparathyroidism	1M	58	NA	NA		✓	Typical PD features. Very fast disease progression.
Pestana Knight, E.M., Gilman, S. & Selwa, L., 2009. [45]	Case report	Wilson’s Disease	1M	55	NA	NA		✓	Typical PD features associated with epilepsy.
Racette, B.A. et al., 2001. [15]	Case report	PKAN	1F	60	NA	NA		✓	Bilateral features, no response to L-dopa.
Rohani, M. et al., 2017. [66]	Case report	Fahr disease	1F	50	NA	NA	✓		Typical L-dopa responsive parkinsonism.
Rosana, A. & La Rosa, L., 2007. [36]	Case report	Hereditary Haemochromatosis	1M	58	NA	NA		✓	No response to L-dopa.
Sakarya, A., Oncu, B. & Elibol, B., 2012. [19]	Case report	PKAN	1M	16	NA	NA		✓	Early severe cognitive impairment, bilateral onset, pyramidal features.
Scale, T. et al., 2014. [67]	Case report	Fahr Disease	1M	62	NA	NA		✓	No response to L-dopa.
Schneider, S.A. et al., 2010. [12]	Case report	Kufor-Rakeb syndrome	1M	16	Pakistan	NA		✓	Associated with dystonia.
Sechi, G. et al., 2007. [43]	Case report	Wilson’s disease	3F	70	NA	NA	✓		Very late onset L-dopa responsive parkinsonism.
Seo, J.-H., Song, S.-K. & Lee, P.H., 2009. [16]	Case report	PKAN	1M	35	NA	NA		✓	No response to L-dopa.
Song, C.-Y. et al., 2017. [68]	Case report	Pseudohypoparathyroidism	1F	52	NA	NA		✓	Very fast disease progression.
Thomas, M., Hayflick, S.J. & Jankovic, J., 2004. [22]	Cross-sectional study	PKAN	14M/8F	35	NA	NA	✓	✓	Typical parkinsonism seen, though clinical features not defined. Associated with dystonia in 4/22 pts.
Vroegindeweij, L.H.P. et al., 2017. [69]	Case Series	Aceruloplasminemia	4M/1F	NA	4 Dutch, 1 Italian	NA		✓	Parkinsonian features in all pts. Associated with cognitive decline and cerebellar features in all pts.
Williams, S. et al., 2013. [37]	Case report	Hereditary Haemochromatosis	1F	60	Caucasian	NA		✓	Short disease course, early autonomic involvement, no L-dopa response.
Xie, F. et al., 2015. [29]	Case report	PLAN	2M	34	NA	NA	✓		Typical features, good L-dopa response.

## References

[1] Lubbe S., Morris H.R. (2014). Recent advances in Parkinson’s disease genetics. J. Neurol..

[2] Kalia L.V., Lang A.E. (2015). Parkinson’s disease. Lancet (Lond. Engl.).

[3] Noyce A.J., Bestwick J.P., Silveira-Moriyama L., Hawkes C.H., Giovannoni G., Lees A.J., Schrag A. (2012). Meta-analysis of early nonmotor features and risk factors for Parkinson disease. Ann. Neurol..

[4] Kieburtz K., Wunderle K.B. (2013). Parkinson’s disease: Evidence for environmental risk factors. Mov. Disord..

[5] Deutschlander A.B., Ross O.A., Dickson D.W., Wszolek Z.K. (2018). Atypical parkinsonian syndromes: A general neurologist’s perspective. Eur. J. Neurol..

[6] Sanderson S., Green A., Preece M.A., Burton H. (2006). The incidence of inherited metabolic disorders in the West Midlands, UK. Arch. Dis. Child..

[7] Waber L. (1990). Inborn errors of metabolism. Pediatr. Ann..

[8] Pietracupa S., Martin-Bastida A., Piccini P. (2017). Iron metabolism and its detection through MRI in parkinsonian disorders: A systematic review. Neurol. Sci. Off. J. Ital. Neurol. Soc. Ital. Soc. Clin. Neurophysiol..

[9] Mochizuki H., Yasuda T. (2012). Iron accumulation in Parkinson’s disease. J. Neural Transm..

[10] Moher D., Liberati A., Tetzlaff J., Altman D.G., Group T.P. (2009). Preferred Reporting Items for Systematic Reviews and Meta-Analyses: The PRISMA Statement. PLOS Med..

[11] Ferreira C.R., Gahl W.A. (2017). Disorders of metal metabolism. Transl. Sci. Rare Dis..

[12] Schneider S., Paisan-Ruiz C., Quinn N., Lees A., MD F., Houlden H., Hardy J., Bhatia K.P. (2010). ATP13A2 mutations (PARK9) cause neurodegeneration with brain iron accumulation. Mov. Disord..

[13] Diaz N. (2013). Late onset atypical pantothenate-kinase-associated neurodegeneration. Case Rep. Neurol. Med..

[14] Lee J.-H., Park J., Ryu H.-S., Park H., Kim Y.E., Hong J.Y., Nam S.O., Sung Y.-H., Lee S.-H., Lee J.-Y. (2016). Clinical Heterogeneity of Atypical Pantothenate Kinase-Associated Neurodegeneration in Koreans. J. Mov. Disord..

[15] Racette B.A., Perry A., D’Avossa G., Perlmutter J.S. (2001). Late-onset neurodegeneration with brain iron accumulation type 1: Expanding the clinical spectrum. Mov. Disord..

[16] Seo J.-H., Song S.-K., Lee P.H. (2009). A Novel PANK2 Mutation in a Patient with Atypical Pantothenate-Kinase-Associated Neurodegeneration Presenting with Adult-Onset Parkinsonism. J. Clin. Neurol..

[17] Lee C.-H., Lu C.-S., Chuang W.-L., Yeh T.-H., Jung S.-M., Huang C.-L., Lai S.-C. (2013). Phenotypes and genotypes of patients with pantothenate kinase-associated neurodegeneration in Asian and Caucasian populations: 2 cases and literature review. Sci. World J..

[18] Mak C.M., Sheng B., Lee H.H., Lau K., Chan W., Lam C., Chan Y. (2011). Young-onset parkinsonism in a Hong Kong Chinese man with adult-onset Hallervorden-Spatz syndrome. Int. J. Neurosci..

[19] Sakarya A., Oncu B., Elibol B. (2012). Panthothenate kinase-associated neurodegeneration (PKAN) presenting with language deterioration, personality alteration, and severe parkinsonism. J. Neuropsychiatry Clin. Neurosci..

[20] Alberca R., Rafel E., Chinchon I., Vadillo J., Navarro A. (1987). Late onset parkinsonian syndrome in Hallervorden-Spatz disease. J. Neurol. Neurosurg. Psychiatry.

[21] Darling A., Tello C., Marti M.J., Garrido C., Aguilera-Albesa S., Tomas Vila M., Gaston I., Madruga M., Gonzalez Gutierrez L., Ramos Lizana J. (2017). Clinical rating scale for pantothenate kinase-associated neurodegeneration: A pilot study. Mov. Disord..

[22] Thomas M., Hayflick S.J., Jankovic J. (2004). Clinical heterogeneity of neurodegeneration with brain iron accumulation (Hallervorden-Spatz syndrome) and pantothenate kinase-associated neurodegeneration. Mov. Disord..

[23] Bozi M., Matarin M., Theocharis I., Potagas C., Stefanis L. (2009). A patient with pantothenate kinase-associated neurodegeneration and supranuclear gaze palsy. Clin. Neurol. Neurosurg..

[24] Schneider S.A., Dusek P., Hardy J., Westenberger A., Jankovic J., Bhatia K.P. (2013). Genetics and Pathophysiology of Neurodegeneration with Brain Iron Accumulation (NBIA). Curr. Neuropharmacol..

[25] Saito Y., Kawai M., Inoue K., Sasaki R., Arai H., Nanba E., Kuzuhara S., Ihara Y., Kanazawa I., Murayama S. (2000). Widespread expression of alpha-synuclein and tau immunoreactivity in Hallervorden-Spatz syndrome with protracted clinical course. J. Neurol. Sci..

[26] Gregory A., Westaway S.K., Holm I.E., Kotzbauer P.T., Hogarth P., Sonek S., Coryell J.C., Nguyen T.M., Nardocci N., Zorzi G. (2008). Neurodegeneration associated with genetic defects in phospholipase A(2). Neurology.

[27] Paisan-Ruiz C., Li A., Schneider S.A., Holton J.L., Johnson R., Kidd D., Chataway J., Bhatia K.P., Lees A.J., Hardy J. (2012). Widespread Lewy body and tau accumulation in childhood and adult onset dystonia-parkinsonism cases with PLA2G6 mutations. Neurobiol. Aging.

[28] Giri A., Guven G., Hanagasi H., Hauser A.-K., Erginul-Unaltuna N., Bilgic B., Gurvit H., Heutink P., Gasser T., Lohmann E. (2016). PLA2G6 Mutations Related to Distinct Phenotypes: A New Case with Early-onset Parkinsonism. Tremor Other Hyperkinet. Mov. (N. Y.).

[29] Xie F., Cen Z., Ouyang Z., Wu S., Xiao J., Luo W. (2015). Homozygous p.D331Y mutation in PLA2G6 in two patients with pure autosomal-recessive early-onset parkinsonism: further evidence of a fourth phenotype of PLA2G6-associated neurodegeneration. Parkinsonism Relat. Disord..

[30] Kim Y.J., Lyoo C.H., Hong S., Kim N.Y., Lee M.S. (2015). Neuroimaging studies and whole exome sequencing of PLA2G6-associated neurodegeneration in a family with intrafamilial phenotypic heterogeneity. Parkinsonism Relat. Disord..

[31] Costello D.J., Walsh S.L., Harrington H.J., Walsh C.H. (2004). Concurrent hereditary haemochromatosis and idiopathic Parkinson’s disease: A case report series. J. Neurol. Neurosurg. Psychiatry.

[32] Girotra T., Mahajan A., Sidiropoulos C. (2017). Levodopa Responsive Parkinsonism in Patients with Hemochromatosis: Case Presentation and Literature Review. Case Rep. Neurol. Med..

[33] Kumar N., Rizek P., Sadikovic B., Adams P.C., Jog M. (2016). Movement Disorders Associated With Hemochromatosis. Can. J. Neurol. Sci..

[34] Nielsen J.E., Jensen L.N., Krabbe K. (1995). Hereditary haemochromatosis: A case of iron accumulation in the basal ganglia associated with a parkinsonian syndrome. J. Neurol. Neurosurg. Psychiatry.

[35] Demarquay G., Setiey A., Morel Y., Trepo C., Chazot G., Broussolle E. (2000). Clinical report of three patients with hereditary hemochromatosis and movement disorders. Mov. Disord..

[36] Rosana A., La Rosa L. (2007). A case of hereditary haemochromatosis in a patient with extrapyramidal syndrome. Blood Transfus..

[37] Williams S., Vinjam M.R., Ismail A., Hassan A. (2013). A parkinsonian movement disorder with brain iron deposition and a haemochromatosis mutation. J. Neurol..

[38] Ferenci P. (2004). Pathophysiology and clinical features of Wilson disease. Metab. Brain Dis..

[39] Lorincz M.T. (2010). Neurologic Wilson’s disease. Ann. N. Y. Acad. Sci..

[40] Czlonkowska A., Litwin T., Dziezyc K., Karlinski M., Bring J., Bjartmar C. (2018). Characteristics of a newly diagnosed Polish cohort of patients with neurological manifestations of Wilson disease evaluated with the Unified Wilson’s Disease Rating Scale. BMC Neurol..

[41] De Gondim F.A., Araujo D.F., Oliveira I.S., Vale O.C. (2014). Small fiber dysfunction in patients with Wilson’s disease. Arq. Neuropsiquiatr..

[42] Oder W., Grimm G., Kollegger H., Ferenci P., Schneider B., Deecke L. (1991). Neurological and neuropsychiatric spectrum of Wilson’s disease: A prospective study of 45 cases. J. Neurol..

[43] Sechi G., Antonio Cocco G., Errigo A., Deiana L., Rosati G., Agnetti V., Stephen Paulus K., Mario Pes G. (2007). Three sisters with very-late-onset major depression and parkinsonism. Parkinsonism Relat. Disord..

[44] Gasca-Salas C., Alonso A., Gonzalez-Redondo R., Obeso J.A. (2017). Coexisting Parkinson’s and Wilson’s Disease: Chance or Connection?. Can. J. Neurol. Sci..

[45] Pestana Knight E.M., Gilman S., Selwa L. (2009). Status epilepticus in Wilson’s disease. Epileptic Disord..

[46] Rizek P., Kumar N., Jog M.S. (2016). An update on the diagnosis and treatment of Parkinson disease. Can. Med. Assoc. J..

[47] (2009). Oxford Centre for Evidence-Based Medicine—Levels of Evidence (March 2009). https://www.cebm.net/2009/06/oxford-centre-evidence-based-medicine-levels-evidence-march-2009/.

[48] Batla A., Adams M.E., Erro R., Ganos C., Balint B., Mencacci N.E., Bhatia K.P. (2015). Cortical pencil lining in neuroferritinopathy: A diagnostic clue. Neurology.

[49] Behrens M.I., Bruggemann N., Chana P., Venegas P., Kagi M., Parrao T., Orellana P., Garrido C., Rojas C.V., Hauke J. (2010). Clinical spectrum of Kufor-Rakeb syndrome in the Chilean kindred with ATP13A2 mutations. Mov. Disord..

[50] Chinnery P.F., Crompton D.E., Birchall D., Jackson M.J., Coulthard A., Lombes A., Quinn N., Wills A., Fletcher N., Mottershead J.P. (2007). Clinical features and natural history of neuroferritinopathy caused by the FTL1 460InsA mutation. Brain.

[51] Crosiers D., Ceulemans B., Meeus B., Nuytemans K., Pals P., van Broeckhoven C., Cras P., Theuns J. (2011). Juvenile dystonia-parkinsonism and dementia caused by a novel ATP13A2 frameshift mutation. Parkinsonism Relat. Disord..

[52] Di Fonzo A., Chien H.F., Socal M., Giraudo S., Tassorelli C., Iliceto G., Fabbrini G., Marconi R., Fincati E., Abbruzzese G. (2007). ATP13A2 missense mutations in juvenile parkinsonism and young onset Parkinson disease. Neurology.

[53] Eiberg H., Hansen L., Korbo L., Nielsen I.M., Svenstrup K., Bech S., Pinborg L.H., Friberg L., Hjermind L.E., Olsen O.R. (2012). Novel mutation in ATP13A2 widens the spectrum of Kufor-Rakeb syndrome (PARK9). Clin. Genet..

[54] Evans B.K., Donley D.K. (1988). Pseudohypoparathyroidism, parkinsonism syndrome, with no basal ganglia calcification. J. Neurol. Neurosurg. Psychiatry.

[55] Fekete R. (2012). Late onset neurodegeneration with brain-iron accumulation presenting as parkinsonism. Case Rep. Neurol. Med..

[56] Fonderico M., Laudisi M., Andreasi N.G., Bigoni S., Lamperti C., Panteghini C., Garavaglia B., Carecchio M., Emanuele E.A., Gian L.F. (2017). Patient Affected by Beta-Propeller Protein-Associated Neurodegeneration: A Therapeutic Attempt with Iron Chelation Therapy. Front. Neurol..

[57] Gore E., Appleby B.S., Cohen M.L., DeBrosse S.D., Leverenz J.B., Miller B.L., Siedlak S.L., Zhu X., Lerner A.J. (2016). Clinical and imaging characteristics of late onset mitochondrial membrane protein-associated neurodegeneration (MPAN). Neurocase.

[58] Hayflick S.J., Kruer M.C., Gregory A., Haack T.B., Kurian M.A., Houlden H.H., Anderson J., Boddaert N., Sanford L., Harik S.I. (2013). Beta-Propeller protein-associated neurodegeneration: A new X-linked dominant disorder with brain iron accumulation. Brain.

[59] Hermann A., Kitzler H.H., Pollack T., Biskup S., Kruger S., Funke C., Terrile C., Haack T.B. (2017). A Case of Beta-propeller Protein-associated Neurodegeneration due to a Heterozygous Deletion of WDR45. Tremor Other Hyperkinet. Mov. (N. Y.).

[60] Ichinose Y., Miwa M., Onohara A., Obi K., Shindo K., Saitsu H., Matsumoto N., Takiyama Y. (2014). Characteristic MRI findings in beta-propeller protein-associated neurodegeneration (BPAN). Neurol. Clin. Pract..

[61] Klysz B., Skowronska M., Kmiec T. (2014). Mitochondrial protein associated neurodegeneration—Case report. Neurol. Neurochir. Pol..

[62] Ni W., Li H.-F., Zheng Y.-C., Wu Z.-Y. (2016). FTL mutation in a Chinese pedigree with neuroferritinopathy. Neurol. Genet..

[63] Nishioka K., Oyama G., Yoshino H., Li Y., Matsushima T., Takeuchi C., Mochizuki Y., Mori-Yoshimura M., Murata M., Yamasita C. (2015). High frequency of beta-propeller protein-associated neurodegeneration (BPAN) among patients with intellectual disability and young-onset parkinsonism. Neurobiol. Aging.

[64] Olgiati S., Dogu O., Tufekcioglu Z., Diler Y., Saka E., Gultekin M., Kaleagasi H., Kuipers D., Graafland J., Breedveld G.J. (2017). The p.Thr11Met mutation in c19orf12 is frequent among adult Turkish patients with MPAN. Parkinsonism Relat. Disord..

[65] Pearson D.W., Durward W.F., Fogelman I., Boyle I.T., Beastall G. (1981). Pseudohypoparathyroidism presenting as severe Parkinsonism. Postgrad. Med. J..

[66] Rohani M., Poon Y.-Y., Naranian T., Fasano A. (2017). SCL20A2 mutation mimicking fluctuating Parkinson’s disease. Parkinsonism Relat. Disord..

[67] Scale T., Lewis C., Hedayat A., Bilal M., Wani M. (2014). Cerebral calcification from Fahr’s disease with co-existing haemochromatosis. Prog. Neurol. Psychiatry.

[68] Song C.-Y., Zhao Z.-X., Li W., Sun C.-C., Liu Y.-M. (2017). Pseudohypoparathyroidism with basal ganglia calcification: A case report of rare cause of reversible parkinsonism. Medicine (Baltim.).

[69] Vroegindeweij L.H.P., Langendonk J.G., Langeveld M., Hoogendoorn M., Kievit A.J.A., Di Raimondo D., Wilson J.H.P., Boon A.J.W. (2017). New insights in the neurological phenotype of aceruloplasminemia in Caucasian patients. Parkinsonism Relat. Disord..

