# Supplementary material for: Parkinson’s Disease and Metal Storage Disorders: A Systematic Review
Source: Brain Sci. 2018 Oct 31;8(11):194. doi: 10.3390/brainsci8110194 (PMC6267486; doi:10.3390/brainsci8110194)
Supplement: Supplementary File 1 [file brainsci-08-00194-s001.zip › Supplementary information/Appendix A. Search terms used for the database searches.pdf]

Term A: Parkinson, Parkinson's, Parkinsonism, Parkinsonism.

Term B: Aceruloplasminemia, Acrodermatitis, Bartter disease, BPAN, Calcium metabolism, CoPAN, Copper metabolism, DiGeorge, Fahr, Hemochromatosis, Hereditary rickets, Iron metabolism, Kufor-Rakeb syndrome, Magnesium metabolism, Menkes, Mitochondrial membrane protein-associated neurodegeneration, Neurodegenerative brain iron accumulation, Neuroferritinopathy, PLAN, PLA2G6-associated neurodegeneration, Phosphate metabolism, Pseudohypoparathyroidism, Tumoral calcinosis, Vitamin D metabolism, Wilson, Woodhouse-Sakati syndrome, Zinc metabolism
