# Supplementary material for: Parkinson’s Disease and Metal Storage Disorders: A Systematic Review
Source: Brain Sci. 2018 Oct 31;8(11):194. doi: 10.3390/brainsci8110194 (PMC6267486; doi:10.3390/brainsci8110194)
Supplement: Supplementary File 1 [file brainsci-08-00194-s001.zip › Supplementary information/Appendix B. Table showing the individual breakdown of the included publications. NA=Not Available..pdf]

Scale, T. et al., 2014. [67]	Case report	Fahr Disease	1M	62	NA	NA		✓	No response to L-dopa
Schneider, S.A. et al., 2010. [12]	Case report	Kufor-Rakeb syndrome	1M	16	Pakistan	NA		✓	Associated with dystonia
Sechi, G. et al., 2007. [43]	Case report	Wilson's Disease	3F	70	NA	NA	✓		Very late onset L-dopa responsive parkinsonism
Seo, J.-H., Song, S.-K. & Lee, P.H., 2009. [16]	Case report	PKAN	1M	35	NA	NA		✓	No response to L-dopa
Song, C.-Y. et al., 2017. [68]	Case report	Pseudohypoparathyroidism	1F	52	NA	NA		✓	Very fast disease progression
Thomas, M., Hayflick, S.J. & Jankovic, J., 2004. [22]	Cross-sectional study	PKAN	14M/8F	35	NA	NA	✓	✓	Typical parkinsonism seen, though clinical features not defined. Associated with dystonia in 4/22 pts
Vroegindeweij, L.H.P. et al., 2017. [69]	Case Series	Aceruloplasminemia	4M/1F	NA	4 Dutch, 1 Italian	NA		✓	Parkinsonian features in all pts. Associated with cognitive decline and cerebellar features in all pts.
Williams, S. et al., 2013. [37]	Case report	Hereditary Haemochromatosis	1F	60	Caucasian	NA		✓	Short disease course, early autonomic involvement, no L-dopa response
Xie, F. et al., 2015. [29]	Case report	PLAN	2M	34	NA	NA	✓		Typical features, good L-dopa response

Nielsen, J.E., Jensen, L.N. & Krabbe, K., 1995. [34]	Case report	Hereditary Haemochromatosis	1M	29	NA	NA	✓		Typical PD features, immediate improvement with L-dopa
Nishioka, K. et al., 2015. [63]	Cross-sectional study	BPAN	7F	32	NA	NA		✓	Cognitive dysfunction as presenting symptom in all 7. Otherwise typical parkinsonism. L-dopa responsive
Oder, W. et al., 1991. [42]	Cross-sectional study	Wilson's Disease	NA	NA	NA	NA	✓		8/25 pts with parkinsonian features. Bradykinesia, resting tremor present.
Olgiati, S. et al., 2017. [64]	Cross-sectional study	MPAN	NA	NA	NA	NA		✓	9/15 pts with parkinsonian features. Cognitive impairment and pyramidal signs seen
Pearson, D.W. et al., 1981. [65]	Case report	Pseudohypoparathyroidism	1M	58	NA	NA		✓	Typical PD features. Very fast disease progression
Pestana Knight, E.M., Gilman, S. & Selwa, L., 2009. [45]	Case report	Wilson's Disease	1M	55	NA	NA		✓	Typical PD features associated with epilepsy
Racette, B.A. et al., 2001. [15]	Case report	PKAN	1F	60	NA	NA		✓	Bilateral features, no response to L-dopa
Rohani, M. et al., 2017. [66]	Case report	Fahr disease	1F	50	NA	NA	✓		Typical L-dopa responsive parkinsonism
Rosana, A. & La Rosa, L., 2007. [36]	Case report	Hereditary Haemochromatosis	1M	58	NA	NA		✓	No response to L-dopa
Sakarya, A., Oncu, B. & Elibol, B., 2012. [19]	Case report	PKAN	1M	16	NA	NA		✓	Early severe cognitive impairment, bilateral onset, pyramidal features.

Hayflick, S.J. et al., 2013. [58]	Cohort study	BPAN	3M/18F	25	NA	NA		✓	Developmental delay, dystonia and parkinsonism. L-dopa responsive
Hermann, A. et al., 2017. [59]	Case report	BPAN	1F	24	German	NA		✓	Supranuclear gaze palsy, dystonia and no L-dopa response
Ichinose, Y. et al., 2014. [60]	Case report	BPAN	1F	30	NA	NA		✓	Associated with dystonia
Kim, Y.J. et al., 2015. [30]	Case Series	PLAN	1M/1F	14	Korean	NA		✓	Associated with dystonia in 2/2 pts.
Klysz, B., Skowronska, M. & Kmiec, T., 2014. [61]	Case report	MPAN	1F	15	NA	NA		✓	Chorea, dystonia and psychological manifestations
Kumar, N. et al., 2016. [33]	Case Series	Hereditary Haemochromatosis	2M/1F	59	1 Irish-Portuguese, 1 Scottish, 1 unknown	NA	✓		Parkinsonian signs in 3 pts. 1 responded well to L-dopa, one not treated.
Lee, C.-H. et al., 2013. [17]	Case report	PKAN	2M	20	Taiwanese	NA	✓	✓	Typical parkinsonism in 1pt though onset at 18. Bilateral features in the other
Lee, J.-H. et al., 2016. [14]	Cross-sectional study	PKAN	6M	36	NA	NA		✓	Poor response to L-dopa in all. Associated with dystonia in 4/6 pts, isolated parkinsonism in 2/6 pts.
Mak, C.M. et al., 2011. [18]	case report	PKAN	1M	27	Hong Kong	NA		✓	Bilateral features
Ni, W. et al., 2016. [62]	Case report	Neuroferritinopathy	1F	44	NA	NA		✓	No response to L-dopa, pyramidal signs

Di Fonzo, A. et al., 2007. [52]	Cross-sectional study	Kufor-Rakeb Syndrome	3M	NA	NA	NA	✓	✓	Features of Parkinsonism in all 3pts. Supranuclear gaze palsy and hallucinations/psychotic episodes in 1/3, psychotic episodes in 1/3 and typical features in 1/3.
Diaz, N., 2013. [13]	Case report	PKAN	1F	NA	NA	NA		✓	L-dopa unresponsive, symmetrical features.
Eiberg, H. et al., 2012. [53]	Case report	Kufor-Rakeb Syndrome	1M	12	NA	NA		✓	Supranuclear gaze palsy, cognitive impairment and hallucinations
Evans, B.K. & Donley, D.K., 1988. [54]	Case report	Pseudohypoparathyroidism	1F	20	NA	NA		✓	Rest tremor and bradykinesia with mental retardation
Fekete, R., 2012. [55]	Case report	NBIA, unknown type	1M	73	NA	NA		✓	Typical features. Poor L-dopa response but dystonia present upon removal of L-dopa.
Fonderico, M. et al., 2017. [56]	Case report	BPAN	1F	26	NA	NA	✓		Mild typical parkinsonism
Gasca-Salas, C. et al., 2017. [44]	Case report	Wilson's Disease	1F	38	NA	NA		✓	Tremor, clumsiness, rigidity and dystonia in left arm. Good L-dopa response.
Giri, A. et al., 2016. [28]	Case report	PLAN	1F	27	NA	NA	✓		Typical Features, PD diagnosis
Girotra, T., Mahajan, A. & Sidiropoulos, C., 2017. [32]	Case report	Hereditary Haemochromatosis	1M	41	Caucasian	NA	✓		Typical features, mild but clear response to L-dopa
Gondim, F. de A.A. et al., 2014. [41]	Case Series	Wilson's Disease	2M/2F	28	Brazil	NA	✓		4 pts with typical features, all responded well to L-dopa
Gore, E. et al., 2016. [57]	Case report	MPAN	1M	35	Kuwaiti	NA		✓	Early behavioural change,

Paper	Type of paper	Condition	Male/Female	Average age at Onset of Parkinsonism (years)	Ethnicity	Smoking Status	Typical Parkinsonism	Atypical Parkinsonism	Parkinsonism features
Alberca, R. et al., 1987. [20]	Case report	PKAN	1M/1F	27	NA	NA	✓	✓	Female siblings: Typical features. Male sibling: associated with dystonia. Fast progression.
Batla, A. et al., 2015. [48]	Case report	Neuroferritinopathy	1F	79	NA	NA		✓	Associated with dystonia
Behrens, M.I. et al., 2010. [49]	Case Series	Kufor-Rakeb Syndrome	4M/1F	NA	Chilean	NA		✓	Parkinsonian features in all 5 pts. No tremor present. Supranuclear gaze palsy in 4/5, poor L-dopa response
Bozi, M. et al., 2009. [23]	Case report	PKAN	1M	15	NA	NA		✓	Mildly affected but associated with pyramidal signs
Chinnery, P.F. et al., 2007. [50]	Cross-sectional study	Neuroferritinopathy	3F	NA	2 English, 1 French	NA		✓	Associated with dystonia in all 3. No tremor present.
Costello, D.J. et al., 2004. [31]	Case report	Hereditary Haemochromatosis	3M/1F	53	NA	NA	✓		4 pts all with HH and IPD diagnoses, classical signs. Good L-dopa response
Crosiers, D. et al., 2011. [51]	case report	Kufor-Rakeb syndrome	1M	10	Afghan	NA		✓	Associated with dystonia.
Czlonkowska, A. et al., 2018. [40]	Cross-sectional study	Wilson's Disease	NA	NA	Polish	NA	✓		Parkinsonism found in 11.3% (6/53pts)
Darling, A. et al., 2017. [21]	Cross-sectional study	PKAN	22M/25F	NA	NA	NA		✓	Features of Parkinsonism displayed in all 47pts. Associated with Dystonia.
Demarquay, G. et al., 2000. [35]	Case report	Hereditary Haemochromatosis	2M/1F	56	NA	NA		✓	Bradykinesia and rigidity on left side. Poor L-dopa response
